# Ruptured Hepatocellular Carcinoma: What Do Interventional Radiologists Need to Know?

**DOI:** 10.3389/fonc.2022.927123

**Published:** 2022-06-16

**Authors:** Jingxin Yan, Ting Li, Manjun Deng, Haining Fan

**Affiliations:** ^1^ Department of Interventional Therapy, Affiliated Hospital of Qinghai University, Xining, China; ^2^ Department of Postgraduate, Qinghai University, Xining, China; ^3^ Department of Orthopedics, Sichuan Provincial People’s Hospital, Chengdu, China; ^4^ Department of Postgraduate, Chengdu Medical College, Chengdu, China; ^5^ Department of Hepatopancreatobiliary Surgery, Affiliated Hospital of Qinghai University, Xining, China; ^6^ Qinghai Province Key Laboratory of Hydatid Disease Research, Xining, China

**Keywords:** rupture of hepatocellular carcinoma, transarterial embolization (TAE), transarterial chemoembolization (TACE), ablation, prognosis, interventional radiology

## Abstract

Rupture of HCC (rHCC) is a life-threatening complication of hepatocellular carcinoma (HCC), and rHCC may lead to a high rate of peritoneal dissemination and affect survival negatively. Treatment for rHCC mainly includes emergency surgery, interventional therapies, and palliative treatment. However, the management of rHCC should be carefully evaluated. For patients with severe bleeding, who are not tolerant to open surgery, quick hemostatic methods such as rupture tissue ablation and TAE/TACE can be performed. We described clinical presentation, prognosis, complication, interventional management, and current evidence of rHCC from the perspective of interventional radiologists. Overall, our review summarized that interventional therapies are necessary for most patients with rHCC to achieve hemostasis, even in some patients with Child–Pugh C. Moreover, TAE/TACE followed by staged hepatectomy is a beneficial treatment for rHCC according to current clinical evidence. TAE/TACE is the first choice for most patients with rHCC, and appropriate interventional treatment may provide staged surgery opportunities for those who are not tolerant to emergency surgery to reach an ideal prognosis.

## Introduction

Hepatocellular carcinoma (HCC) is the sixth most diagnosed cancer and the fourth leading cause of cancer-related death worldwide (1). Hepatitis C virus (HCV) infections and hepatitis B virus (HBV) infections account for most cases of HCC, and the rest mainly comprise people with excessive alcohol consumption, nonalcoholic fatty hepatitis, liver cirrhosis, and a family history of HCC (2, 3). Spontaneous rupture of HCC (rHCC) is a life-threatening complication with an estimated incidence of 3%–26% in patients with HCC (4, 5) and a mortality rate ranging from 25% to 75% (6–9). Furthermore, even if the hemorrhage is completely controlled, rHCC may lead to a high rate of peritoneal dissemination and affect patient survival negatively (10). Therefore, rHCC is significantly associated with high risk of recurrence and poor prognosis (11). The primary aim remains the prevention of hypovolemic shock and the stabilization of the condition of patients (12, 13); thus, therapeutic options should be discussed according to the condition of the patient, tumor stage, liver function, and feasibility of resection. The treatment options for spontaneous rHCC mainly include emergency surgery, interventional therapy, and palliative treatment. However, the choice of emergency surgery is still controversial because some studies believed that patients with spontaneous rHCC should be managed by non-operative treatment initially (14). Moreover, emergency surgery has a high risk for elderly patients or patients with poor physical condition (15, 16). Emergency surgery may increase the risk of intraperitoneal hemorrhage, liver failure, abdominal infection, and bile leakage. According to the American Joint Committee on Cancer, all patients with spontaneous rHCC are assigned a grade of T4, even if the tumor is small, and there is no vascular invasion (17), indicating the poor prognosis of rHCC; thus, whether those with rHCC are tolerant to emergency surgery should be carefully evaluated (18, 19). Another controversial point is that emergency surgery may prolong the overall survival time for some patients, but it may increase the risk of tumor metastasis for others (18, 20). After the first 12 h of clinical management without significant improvement, supportive care could be chosen in situations of acute uncontrolled bleeding in moribund patients (21). Initial management of rHCC includes a multidisciplinary and comprehensive approach with a primary aim of patient survival, rather than HCC treatment.

Interventional therapy, including transarterial chemoembolization (TACE), transarterial embolization (TAE), transarterial radioembolization (TARE), microwave ablation (MWA), and radiofrequency ablation (RFA), remains a minimally invasive treatment for HCC, even suitable for some patients who have extrahepatic metastases or recurrence (4). TACE/TAE could embolize the blood supply of rHCC *via* the hepatic artery and other extrahepatic arteries. The application of interventional therapy is still broader than open surgery, as interventional therapy can be applied in some cases with Child–Pugh C (22). Furthermore, for patients with severe bleeding, who are not tolerant to open surgery, quick hemostatic methods such as rupture tissue ablation and TAE/TACE can be performed (23). This review will aim to clarify the clinical presentation, prognosis, complication, interventional management, and current evidence of rHCC from the perspective of interventional radiologists.

## Pathophysiology and Risk Factors of HCC Rupture

The mechanism and prognosis of rHCC have not been completely investigated. Studies have also reported that tumor location, tumor size, hypertension, hepatic parenchyma, and liver cirrhosis were associated with rHCC.

The overlying liver parenchyma plays an important role in rHCC. As tumor progresses, when the tumor invades the hepatic arteries or portal vein, the pressure inside the liver may increase and lead to rHCC (24–26). Moreover, a study found that there was almost no overlying liver parenchyma on the ruptured tumor, neither was it shown by imaging examination nor found in operation (27).

The relationship between tumor size and HCC rupture is controversial. Although the size of tumor is associated with the pressure inside the tumor and vessel, Li et al. (27) reported that tumor size is not a significant risk factor in rHCC. However, Chen et al. (25) and Zhu et al. (28) reported that HCC size of more than 5 cm is at high risk of rHCC. Undoubtedly, tumor size is an independent prognostic factor in evaluating overall survival and disease-free survival (29, 30). If the mass volume increases too fast and partial necrosis of the tumor occurs, it can lead to the collapse of the tumor surface and bleeding. Furthermore, researchers also found that an HCC as small as 2 cm has been reported to be ruptured (31). Tumor size is an important study-level factor of rHCC, but clinicians cannot predict rHCC simply by tumor size.

Previous studies have identified that vascular injury may be associated with rHCC. A widely accepted mechanism of rHCC is that hepatic arteries and veins are invaded or occluded by tumor cells, leading to high pressure within the tumor mass (32). As HCC is supplied by the tumor supply vessels, Zhu et al. (33) reported that the vascular injury was present more frequently in the patients with rHCC than that in the patients with non-ruptured HCC. Moreover, Zhu et al. (34) reported that the expression of the antigen–antibody complex including hepatitis B e1 antigen and complement C1q on vascular wall and vascular injuries was mainly presented in patients with rHCC. Their conclusion can also explain why it is more common to see patients with rHCC in Asia than in Europe, because Asia has the highest incidence rates of chronic HBV infection (especially in China) (35). In addition, due to the invasion and growth of tumor, the pressure of hepatic vein may increase, and the high pressure of hepatic vein may be associated with venous congestion, resulting in tumor ischemia, necrosis, and liquefaction. Hibi (36) reported that increased intraluminal pressure of the biliary system due to obstruction by the tumor thrombus is considered to have led to the rupture of the liver abscess into the bronchus, thus creating a bronchobiliary fistula; thus, intraluminal pressure of the biliary system may be associated with a series of risks including tumor rupture. As patients with HCC can be affected by various factors such as coagulopathy and abnormal liver function, all these factors lead to rHCC and intrableeding of tumor (12).

In addition, rHCC is associated with factors involving liver function, portal vein thrombus, location, and so on. Monroe et al. (37) reported that patients with a higher degree of liver dysfunction have an overall poor prognosis and a high possibility of rHCC. After the portal vein was embolized by tumor thrombus, dystrophic necrosis appeared in the peripheral part of the superficial tumor, and the rupture of superficial tumor may also be associated with rHCC (38); thus, the location of the tumor is a risk factor for rHCC. On the basis of the experience from our center, when the tumor is located at the superficial position of the hepatic encapsulation, it can be easily affected by external forces. The thin tumor encapsulation and the extremely fragile cancer tissue also cause rupture and bleeding.

## Clinical Presentations and Diagnosis of Ruptured HCC

Although rHCC can present with various symptoms, acute abdominal pain can be observed in most cases (66%–100%) of rHCC (17). Shock is the second most common complication of rHCC, with an incidence range from 33% to 90% (7, 9, 20, 39–41). Liver failure occurs in 12%–42% of rHCC (12). Furthermore, in the study by Zhu et al. (28), patients with spontaneous rHCC can be asymptomatic, or experience abdominal discomfort and anemic symptoms, indicating that the presentation of rHCC varies from individuals. Even in some rare cases, patients presented with non-bleeding rHCC (42) or hemothorax (43).

Ultrasonography (US) and computed tomography (CT) are recommended for detecting rHCC, the location of ruptured tumor, and changes in hematoma density. The most direct evidence of rHCC from CT scans is hemoperitoneum (44), but hemoperitoneum cannot be observed in all cases. Moreover, highest-attenuating hematomas, active extravasation of contrast materials, contour protrusion of tumor, discontinuity of the hepatic surface, and the enucleation sign are various CT findings of usual manifestations of rHCC (25, 45–49). Furthermore, the location of highest-attenuation hematomas is close to the rHCC (50).

Patients with rHCC usually presented with acute abdominal pain, and with the utilization of medical imaging, laboratory analyses, and history of patients, a diagnosis of rHCC can usually be made. However, in some cases of rHCC, the diagnosis of rHCC is still a challenge, as some cases are noncirrhotic and there is an absence of risk factors, indicating that other potential factors may also play an important role in carcinogenesis and rHCC (51, 52).

Abdominal paracentesis can be a choice when patients are suspected with rHCC (53). If the non-coagulable blood was sucked out by a syringe, it shows that there is visceral hemorrhage in the abdominal cavity, which is the most effective and routine examination method for the diagnosis of abdominal visceral hemorrhage (54). However, the coagulation function of HCC patients should be taken into account when abdominal paracentesis is performed. Moreover, diagnostic paracentesis is in vain when performed in some rare case presented with no bleeding (42). It is worth noting that no diagnostic study has been published to investigate the sensitivity and specificity of abdominal paracentesis for the diagnosis of rHCC.

## Transarterial Embolization/Transarterial Chemoembolization for Rupture of HCC

Although the prognosis of rHCC is not ideal, survival benefits for interventional radiology and hepatectomy have been reported in patients with rHCC (55–57). Actually, several studies (55, 58, 59) reported that hepatectomy provided better survival benefits than TAE/TACE, reaching an in-hospital survival of 76.5% and a 1-month survival of 71%. However, TAE is the most frequent option to achieve hemostasis and stabilize the condition of patients. The hemostatic success rate from published studies is ideal as most patients can achieve hemostasis (**Table 1**) (29, 30, 58–79). A meta-analysis (5) involving 21 studies with 974 rHCC participants (485 participants treated with TACE/TAE and 489 participants treated with emergency surgery) reported that TAE/TACE was significantly superior to emergency surgery in terms of complications [OR = 0.36, 95% confidence interval (CI): 0.22–0.57] and in-hospital mortality (OR = 0.52, 95% CI: 0.29–0.94), and emergency surgery did not provide a more favorable effect on successful hemostasis (96.2% vs. 94.6%) and 1-year survival rate (47.6% vs. 48.7%) than TAE/TACE. However, all included studies of this meta-analysis were conducted in China, which will undoubtedly lead to bias. Another meta-analysis and systematic review regarding rHCC reported that among patients treated with TACE/TAE, the in-hospital and 1-month survival ranged from 30.3% to 66.7% and from 44.4% to 87.5% (80). Furthermore, Zhou et al. (59) reported that there are no differences in successful rate of hemostasis between TAE and hepatectomy. Furthermore, due to the abnormal coagulation function, poor hepatic function reserve, hemodynamic instability, cirrhosis, and other risk factors (58, 80, 81), many patients with rHCC are not well tolerant to open surgery. Interventional therapies involving TAE/TACE for rHCC can reach an ideal effect. TACE/TAE can be performed as a temporary treatment for patients waiting for staged hepatectomy. Even some cases with poor prognostic factors such as liver function deteriorated, and aforementioned interventional options may be employed in patients with hemodynamic instability or advanced underlying patients with rHCC.

**Table 1:** Summary of clinical studies on successful hemostasis, survival, and embolization agents of TAE/TACE for rHCC.

Study	Study type	Number of patients	Etiology	Intervention	Successful hemostasis	30-day survival	1-year survival	Embolization agents
Bakopoulos A 2018 (60)	Case report	1	HBV	TAE	1	1	1	Microspheres
Barah A 2018 (61)	Case series	2	NA	TAE	2	NA	NA	Gelatin sponge
Battula N 2009 (29)	Case series	7	NA	TAE	5	NA	NA	NA
Buczkowski AK 2005 (62)	Retrospective study	16	NA	TAE	13	NA	NA	A variety of embolic agents were used
Cheng Y 2021 (63)	Retrospective study	186	HBV: 83 HCV: 53 B+C/: 5 Non-B, non-C: 45	TAE	170	148	76	Lipiodol
Kirikoshi H 2009 (30)	Retrospective study	16	HBV: 11 HCV: 1 Alcohol: 1 Non-B, non-C: 3	TAE	15	14	4	NA
Lee HS 2019 (64)	Retrospective study	112	NA	TACE/TAE	107	NA	NA	NA
Masumuto A 1997 (65)	Case report	1	HCV	TAE	1	1	0	NA
Shinmura K 2018 (66)	Retrospective study	51	Alcohol:6 HBV: 21 HCV: 14 Non-B, non-C: 5	TAE	NA	32	NA	Gelatin sponge
Qiu Y 2021 (67)	Retrospective study	322	HBV: 94	TAE	NA	NA	152	NA
Lee K 2019 (68)	Retrospective study	118	HBV: 49 Non-HBV: 69	TAE	118	66	NA	Gel foam slurry and/or PVA
Hsueh K 2012 (69)	Retrospective study	29	NA	TAE	NA	26	18	NA
Kodama Y 2002 (70)	Retrospective study	1	NA	TAE	1	1	NA	Gelatin sponge
Miyayama S 2001 (71)	Case series	3	NA	TAE	3	NA	NA	Lidocaine
Yang P 2007 (72)	Case report	1	HBV	TAE	1	1	1	Coil
Zhou C 2018 (73)	Retrospective study	57	HBV: 47 Other: 12	TAE	57	49	21	Lipiodol or gelatin sponge
Zhou C 2020 (74)	Retrospective study	20	HBV: 20	TAE	20	NA	16	Lipiodol or gelatin sponge particles or PVA
Shin BS 2010 (75)	Retrospective study	47	HBV: 29 HCV: 4 Non-B, non-C: 17	TACE/TAE	44	35	20	Lipiodol or gelatin sponge particles or PVA
Shiozawa K 2013 (76)	Case report	1	Alcohol-related cirrhosis	TAE	1	NA	NA	Sponge particles
Yang H 2014 (58)	Retrospective study	41	NA	TAE	NA	30	4	NA
Yoshiya S 2018 (77)	Case report	1	Non-B	TAE	1	1	NA	Sponge particles
Zhang D 2015 (78)	Retrospective study	126	Alcohol:3 HBV: 114 HCV: 3 Negative: 6	TAE	126	121	99	Sponge particles
Zhong F 2016 (79)	Retrospective study	48	HBV: 41 HCV: 6 Other: 1	TAE	48	39	NA	Gel foam
Zhou C 2020 (59)	Retrospective study	59	NA	TAE	59	32	NA	Lipiodol or gelatin sponge particles or PVA

PVA, polyvinyl alcohol particles; TAE, transarterial embolization; TACE, transarterial chemoembolization; NA, not available.

The aim of interventional therapy is not only to achieve successful hemostasis, but also to prolong the survival of patients with rHCC. Lee et al. (64) and Hsueh et al. (69) reported that those who underwent staged hepatectomy after TACE/TAE had significantly higher overall survival than those who underwent TACE alone, indicating that staged hepatectomy is encouraged for post-TAE/TACE patients who are tolerant to hepatectomy. However, whether TAE/TACE is to be followed by staged hepatectomy depends on the recovery of liver function and a thorough investigation of the tumor characteristics. Moreover, whether HCC is ruptured, staged hepatectomy is recommended (12, 20, 82). It should be noted that a meta-analysis (83) reported that long-term staged hepatectomy might not be more beneficial than emergency hepatectomy. However, in this meta-analysis, we found that the authors did not mention how they acquire survival data to estimate hazard ratio from included studies and their results contradict the studies that they included; thus, their conclusion should be interpreted with caution.

Chen et al. (38) and Kirikoshi et al. (30) reported that the main cause of death is failed hemostasis after TAE and hepatic failure. Furthermore, Cheng et al. (63) reported that Child–Pugh classification, MELD score, BCLC stage, albumin, INR, and post-TAE total bilirubin in 7 days are independent risk factors for post TAE 30-day mortality and successful hemostasis in rHCC.

Interventional radiologists should also note that extrahepatic rupture of metastatic HCC has been reported as a rare complication of TAE (84). Suoh et al. (85) reported a case of hemothorax secondary to spontaneous rHCC metastasis to the chest wall in an 87-year-old man who was treated with TAE, and transcatheter treatment can achieve hemostasis and a favorable survival even in this setting. Nagao et al. (86) reported a case of ruptured chest wall metastasis of HCC that was controlled by TAE.

Kodama et al. (70) reported of a case with rHCC supplied by the right renal capsular artery and Child–Pugh C liver function, indicating that TAE may be chosen for poor liver function when tumor feeders are only extrahepatic collateral vessels. However, Bassi et al. (87) reported that the mortality of patients with Child–Pugh C liver function is exceedingly high in the early post-TAE period, but only three patients with Child–Pugh C liver function received TAE in this study. TAE is a palliative procedure that is used when the liver function is compromised or, in the case of multifocal-bilobar HCC, when the post-treatment mortality of poor liver function is very high regardless of treatment type. In Shin’s study (75), the overall median survival time of ten patients with Child–Pugh class C who underwent TAE/TACE was 5 days.

HCC can recruit extrahepatic collateral vessels from a wide range of vessels such as gastroduodenal artery, superior mesenteric artery, and suprarenal arteries (88–90); thus, incomplete embolization of those vessels may also raise the risk of rebleeding. The estimated prevalence of extrahepatic arteries recruited by HCC ranged from 17% to 27% (91–94), indicating that some cases with extrahepatic collateral arteries cannot be successfully embolized because those vessels may originate from other organs such as stomach and duodenum. Moreover, due to the incomplete embolization of extrahepatic collateral arteries, recurrent bleeding and local tumor recurrence should be noted by interventional radiologists in clinical practice. In rHCC with celiac axis stenosis, Barah et al. (61) reported that the pancreaticoduodenal was used as a salvage alternative route for emergency TAE of hepatic arteries. However, in situations such as extensive stenosis or occlusion of the origin of the celiac axis, inaccessibility to hepatic arteries through the celiac axis is a highly challenging situation in which the technical success depends on the experience of interventional radiologists.

It is worth noting that TAE/TACE-led injuries of portal vein or hepatic vein may lead to recurrent bleeding and an unsatisfactory hemostatic effect (95, 96), as HCC can be ruptured after TAE/TACE or during TAE/TACE (97). Furthermore, some patients are at high risk of rupture because of a lack of protective surrounding parenchymal tissue, or tumor progression resulting from revascularization after embolization (98). Moreover, TACE may lead to a series of post-chemoembolization syndrome including pain and fever. Most patients who underwent chemoembolization may experience post-chemoembolization syndrome, but the data are lacking. Moreover, TACE may also be related to acute renal failure, upper gastrointestinal bleeding, and other serious complications (99–101). This may be the reason why TAE is much more frequently performed for patients with rHCC than TACE. Interestingly, Hidaka et al. reported that TACE for those with huge HCC (>10 cm) may also lead to rHCC (102), and a post-TACE huge HCC rupture can be successfully treated using interventional procedures. Tu et al. (103) reported that liver rupture can be observed in 6 of 1,120 patients who received conventional TAE/TACE therapy. According to aforementioned studies, we infer that patients with a history of TACE/TAE or a tumor diameter that is greater than 10 cm may have a higher incidence of rHCC. However, this hypothesis should be verified by further studies.

Various embolization materials can influence the patient prognosis, and the effect of different embolic agents is not completely known. Koçyiğit et al. (104) reported that the use of different embolic agents for TACE had no significant effect on survival on patients with HCC. Marelli et al. (105) reported that there is no evidence of the benefit of lipiodol, and gelatin sponge is the most used embolic agent, but PVA particles may be better. However, in clinical practice, in general, a variety of embolic agents were used, depending on tumor location, degree of bleeding, and interventional radiologist experience. Furthermore, no study has investigated the effect and safety of different embolic agents in the treatment of rHCC.

Discussing the level of embolization, Lee et al. (68) reported that selective or subsegmental TAE was performed if the tumor is solitary and/or a bleeding source was well delineated by CT and/or angiography. In our opinion, the primary aim remains the prevention of hypovolemic shock and stabilization of the condition of patients, and the secondary aim is to embolize the tumor as much as possible when feasible.

Another issue that should be noted is that aforementioned observational studies, non-randomized studies, case reports, and cluster-randomized trials have a known limitation as these studies will force us to focus more on benefits than harms (106).

Furthermore, the overall survival rate of patients with rHCC who underwent TAE/TACE is lower than those who underwent radical resection; hence, the long-term effect of TAE/TACE is still questioned (12).

## Ablation for Rupture of HCC

Ablation is another interventional therapy for rHCC, but the efficacy and safety are only reported in a few studies (**Table 2**) (107–111). Although ablation involves radiofrequency ablation therapy (RFA), cryoablation, MWA, and irreversible electroporation (IRE), RFA is the main choice in the treatment of HCC, which can be performed using a percutaneous, laparoscopical, or laparotomic approach. However, the safety of the percutaneous approach is not clear, which is associated with the risk of intrahepatic bleeding, bile leakage, and liver injury (108); thus, Livraghi et al. (112) suggested that the open or laparoscopic method is a safer approach in the treatment of subcapsular tumors.

**Table 2:** Summary of clinical studies on successful hemostasis and survival of ablation for rHCC.

Study	Study type	Number of patients	Etiology	Intervention	Successful hemostasis	30-day survival	1-year survival
Cheung T 2014 (107)	Case series	119*	HBV: 86	RFA	NA	93	NA
Gao J 2016 (108)	Case series	6	HBV: 6	Laparoscopic RFA	6	6	6
Warren YE 2016 (109)	Case report	1	NA	MWA	1	1	NA
Huang J 2017 (110)	Case report	1	NA	RFA	1	1	1
Bertacco A 2017 (111)	Case report	1	Alcoholic-related cirrhosis	RFA	1	NA	NA

*Only 19 patients received ablation.

RFA, radiofrequency ablation; MWA, microwave ablation; NA, not available.

Gao et al. (108) reported 10 patients [size: 6.6 ± 2.2 cm (4.0–10.1 cm)] with rHCC treated with laparoscopic RFA, with a 3-year survival rate of 70%. Sasaki et al. (113) reported that ruptured peritoneal metastases of HCC can also be controlled by RFA. Sun et al. (114) reported a case of a giant HCC with a maximum diameter of 14 cm with unstable circulation (Child–Pugh C). During the emergency laparotomy, the authors found that surgical excision was impossible due to the size and location of the tumor. Then, the patient received RFA as a salvage solution. Finally, patient follow-up for at least 56 months revealed a high quality of life, indicating that a giant rHCC can be successfully controlled by RFA. Chen et al. (115) reported that RFA can also be performed in a case of liver metastasis from rectal cancer and a case of liver metastasis from gastric sarcoma, reaching a beneficial survival time of 36 months and 17 months, respectively.

Among these studies, image-guided percutaneous ablation is commonly used, as it can be minimally invasive, and most cases can achieve hemostasis. Because only limited cases were reported, the benefit of ablation is not completely understood. Three studies (107, 108, 110) reported that direct puncturing of the bleeding site was performed. Similar to TAE/TACE, the primary aim of ablation remains the prevention of hypovolemic shock and the stabilization of the condition, and the secondary aim is to achieve tumor elimination when feasible.

Furthermore, another issue of ablation that should be noted is the “heat sink” effect, which is defined as a phenomenon whereby flowing blood adjacent to or within tissues being targeted for ablation results in relative tissue cooling due to heat transfer by convection (116), leading to a bad prognosis. On the other hand, rHCC usually has a large volume and rich blood supply; thus, the “heat sink” effect may have a significant impact on RFA for a ruptured giant HCC. Interestingly, several studies found that the “heat sink” effect has a significant impact on RFA but not MWA in combination with embolization, indicating that the “heat sink” effect plays a more important role in RFA than in MWA (117–119). Puza et al. (120) reported that the “heat sink” effect appears to have a more minor impact on MWA in a rabbit model and the effect of ablation can be affected when performed adjacent to major vessels. On the basis of these studies, we infer that RFA is much more sensitive in the treatment of rHCC with a smaller diameter and MWA is the optimal ablation for a huge rHCC. However, the conclusion is limited because only several well-documented cases are reported.

## Interventional Combination Therapies for Rupture of HCC

The application of interventional combination therapies is used in some settings. However, interventional combination therapies for rHCC are only reported in several studies (**Table 3**) (121, 122). Baimas-George et al. (121) reported that TAE or TACE followed by laparoscopic MWA and washout may offer an advantage in the treatment of rHCC. It not only achieves hemostasis but also could have an oncologic benefit by targeting local tumor and decreasing peritoneal carcinomatosis risk, reaching a median survival of 431 days in 15 patients, but the 30-day mortality was 6/15. Takao et al. (122) reported that a case of hemobilia from HCC can be controlled by TAE followed by MWA; the patient died of liver failure with no recurrence of hemobilia. However, the prognosis of patients who received TAE/TACE followed by MWA should be evaluated by more high-quality clinical evidence. It is too early to draw final conclusions about the impact of interventional combination therapies on rHCC.

**Table 3:** Summary of clinical studies on successful hemostasis, survival, and embolization agents of interventional combination therapies for rHCC.

Study	Study type	Number of patients	Etiology	Intervention	Successful hemostasis	30-day survival	1-year survival	Embolization agents
Gao J 2016 (108)	Case series	4	HBV: 4	TAE followed by laparoscopic RFA	4	4	3	Lipiodol and gelatin sponge
Baimas-George M 2020 (121)	Retrospective study	9	NA	TACE/TAE followed by MWA	NA	NA	7	NA
Takao Y 2008 (122)	Case report	1	HCV	TAE followed by MWA	1	1	0	NA

RFA, radiofrequency ablation; MWA, microwave ablation; NA, not available.

Recently, the interventional combination therapies for HCC include TACE plus brachytherapy and transarterial ethanol ablation combined with TACE (123–125). Moreover, immune checkpoint inhibitors and tyrosine kinase inhibitors are adopted in clinical practice, but there is no evidence to investigate the relationship between rHCC and these drugs (126). Although such treatments are not reported in the treatment of rHCC, those studies prompted us to consider that interventional combination therapies may improve the prognosis of patients with rHCC in the future.

## Conclusion

In conclusion, the clinical management of rHCC remains a challenge. Our review summarized that interventional therapies are necessary for most patients with rHCC to achieve hemostasis, even in some patients with Child–Pugh C. Furthermore, TAE/TACE followed by staged hepatectomy is the most beneficial treatment for rHCC according to current clinical evidence. However, the efficacy and safety of ablation are still questioned because only a few well-documented studies were reported. Interventional radiologists should also keep in mind that TAE/TACE is the first choice for most patients with rHCC, and appropriate interventional treatment may provide staged surgery opportunities for those who are not tolerant to emergency surgery to reach an ideal prognosis.

## Author Contributions

All authors listed have made a substantial, direct, and intellectual contribution to the work and approved it for publication.

## Funding

This study was supported by Project of Science and Technology Department of Qinghai Province (2020-ZJ-Y01).

## Conflict of Interest

The authors declare that the research was conducted in the absence of any commercial or financial relationships that could be construed as a potential conflict of interest.

## Publisher’s Note

All claims expressed in this article are solely those of the authors and do not necessarily represent those of their affiliated organizations, or those of the publisher, the editors and the reviewers. Any product that may be evaluated in this article, or claim that may be made by its manufacturer, is not guaranteed or endorsed by the publisher.
